# Use of a Capture-Based Pathogen Transcript Enrichment Strategy for RNA-Seq Analysis of the *Francisella Tularensis* LVS Transcriptome during Infection of Murine Macrophages

**DOI:** 10.1371/journal.pone.0077834

**Published:** 2013-10-14

**Authors:** Zachary W. Bent, David M. Brazel, Mary B. Tran-Gyamfi, Rachelle Y. Hamblin, Victoria A. VanderNoot, Steven S. Branda

**Affiliations:** Sandia National Laboratories, Livermore, California, United States of America; Cornell University, United States of America

## Abstract

*Francisella tularensis* is a zoonotic intracellular pathogen that is capable of causing potentially fatal human infections. Like all successful bacterial pathogens, *F. tularensis* rapidly responds to changes in its environment during infection of host cells, and upon encountering different microenvironments within those cells. This ability to appropriately respond to the challenges of infection requires rapid and global shifts in gene expression patterns. In this study, we use a novel pathogen transcript enrichment strategy and whole transcriptome sequencing (RNA-Seq) to perform a detailed characterization of the rapid and global shifts in *F. tularensis* LVS gene expression during infection of murine macrophages. We performed differential gene expression analysis on all bacterial genes at two key stages of infection: phagosomal escape, and cytosolic replication. By comparing the *F. tularensis* transcriptome at these two stages of infection to that of the bacteria grown in culture, we were able to identify sets of genes that are differentially expressed over the course of infection. This analysis revealed the temporally dynamic expression of a number of known and putative transcriptional regulators and virulence factors, providing insight into their role during infection. In addition, we identified several *F. tularensis* genes that are significantly up-regulated during infection but had not been previously identified as virulence factors. These unknown genes may make attractive therapeutic or vaccine targets.

## Introduction

*Francisella tularensis*, the causative agent of tularemia, is a Gram-negative facultative intracellular pathogen that is capable of infecting a wide variety of hosts, including mammals, birds, amphibians, fish, and insects [1]. Originally isolated in 1911 in Tulare County, California during a plague-like outbreak in the rodent population, *F. tularensis* was subsequently found to be endemic in most of the northern hemisphere [2]. Human infections most commonly occur upon contact with infected animals or from the bite of an infected tick, leading to cutaneous ulceroglandular tularemia [3]. A pneumonic infection can result from inhalation of as few as 10 bacteria, leading to severe and often fatal disease [4]. Because of the seriousness of its disease, ability to be aerosolized, and extremely low infectious dose, *F. tularensis* has long been feared for its potential as a biological weapon and has been designated a Category A Select Agent [5,6]. The species *F. tularensis* is comprised of two sub-species types, with type A strains endemic to North America and type B strains endemic to Europe and Asia [2]. A live vaccine strain (LVS) derived from a type B strain was created in the former Soviet Union over 50 years ago; due to safety concerns, however, it is not currently licensed for human use [7]. Although the *F. tularensis* LVS strain does not cause illness in humans, it is lethal to mice, causing a disease that very closely mimics human tularemia [8]. These features have made the *F. tularensis* LVS murine infection model an ideal and well-established system for study of *F. tularensis* pathogenesis [9].

As an intracellular pathogen, *F. tularensis* must adapt to multiple environments throughout the course of an infection. The bacteria enter host cells *via* phagocytosis, escape the phagosome, replicate within the host cell cytosol, and at later stages of infection are found within a double membranous compartment that resembles an autophagosome [10]. The bacteria infect a variety of cell types in a variety of locations throughout the body, each presenting different stresses and challenges to bacterial survival [7,11]. The bacteria must appropriately respond to each of these microenvironments for the infection to proceed. To accomplish this, *F. tularensis* must rapidly alter transcription of numerous genes in a coordinated manner as it moves from site to site within the host, as well as within the compartments of individual host cells. Typically, pathogens respond to infection-associated stresses through up-regulation of virulence factors - genes that have been demonstrated by mutational or genetic analysis to play a critical role in the bacteria’s ability to cause disease. These genes encode a wide range of products, including secretion systems, adhesins, invasins, iron uptake pathways, and toxins. During infection pathogens also down-regulate expression of transcripts that are no longer necessary, or are potentially detrimental, in a given microenvironment. These changes in gene expression are ultimately responsible for the success of the pathogen in evading or subverting the immune response and surviving within its host.

Previous work to characterize *F. tularensis* transcriptome dynamics during infection has focused on the type A strain SCHU S4. Wehrly and colleagues used microarray analysis to track the transcriptional profiles of the bacteria during infection of murine bone marrow-derived macrophages (BMDM) [12]. Walters and colleagues used RNA-Seq to investigate the transcriptome of the bacteria at late time points in the lungs of infected mice [13]. Both studies revealed up-regulation of known and previously unknown virulence factors, demonstrating that distinct stages of *F. tularensis* infection are accompanied by global changes in transcriptional profile.

Given the importance of these global transcriptional shifts to the virulence and persistence of the bacteria within the host, it is critical to understand when and how these shifts occur. Using RNA-Seq to address this issue presents a technical challenge, because the vast majority of transcripts present in infected cells are derived from the host, rather than from the bacteria of interest. In this study, we use a newly developed capture-based bacterial transcript enrichment strategy [14] to obtain enough pathogen reads , despite the host-dominated background, to analyze the complete transcriptome of *F. tularensis* LVS during phagosomal escape and cytosolic replication within murine macrophages. Comparison of the transcriptional profiles of the bacteria at these two distinct time points, relative to that of the bacteria in culture, revealed up-regulation of numerous known virulence factors as well as many genes with unknown function that play, as yet, undetermined roles in *F. tularensis* virulence. Further, by analyzing the expression of the known and putative transcriptional regulators encoded by the *F. tularensis* genome, we were able to identify pathways and products that are important at each stage of infection. 

## Results

### *F. tularensis* transcriptional changes during infection

*F. tularensis* bacteria grown to exponential phase in a rich medium, such as the one used in this study, are highly invested in replication, and therefore would be expected to express genes associated with metabolic functions and cell division. Switching from culture in a rich medium to an active infection of host cells presents a specific series of challenges to the bacteria that are addressed through global transcriptional changes. The first phase of *F. tularensis* infection requires that the bacteria be taken up into the host cell by phagocytosis. This is followed by escape from the phagosome, and establishment of a replicating population within the cytosol of the host cell. The exact timing of these events varies in different *F. tularensis* strains, host cell types, and infection protocols. However, it is well documented that in the murine macrophage cell line J774A.1, as well as in murine bone marrow derived macrophages (BMDM), after 4 hours of infection the bacteria are in a transition state in which some are still within phagosomes while others have managed to escape into the cytosol [10,15-18]. After 8 hours the vast majority of bacteria are located within the cytosol [10,12,15,18-20]. Work by Mack and colleagues [21] as well as Edwards and colleagues [22] has demonstrated, through direct comparisons, that *F. tularensis* infections show an essentially identical disease progression in J774A.1 cells as P388D1 cells, the murine macrophage line used in this study.

**Figure 1:**
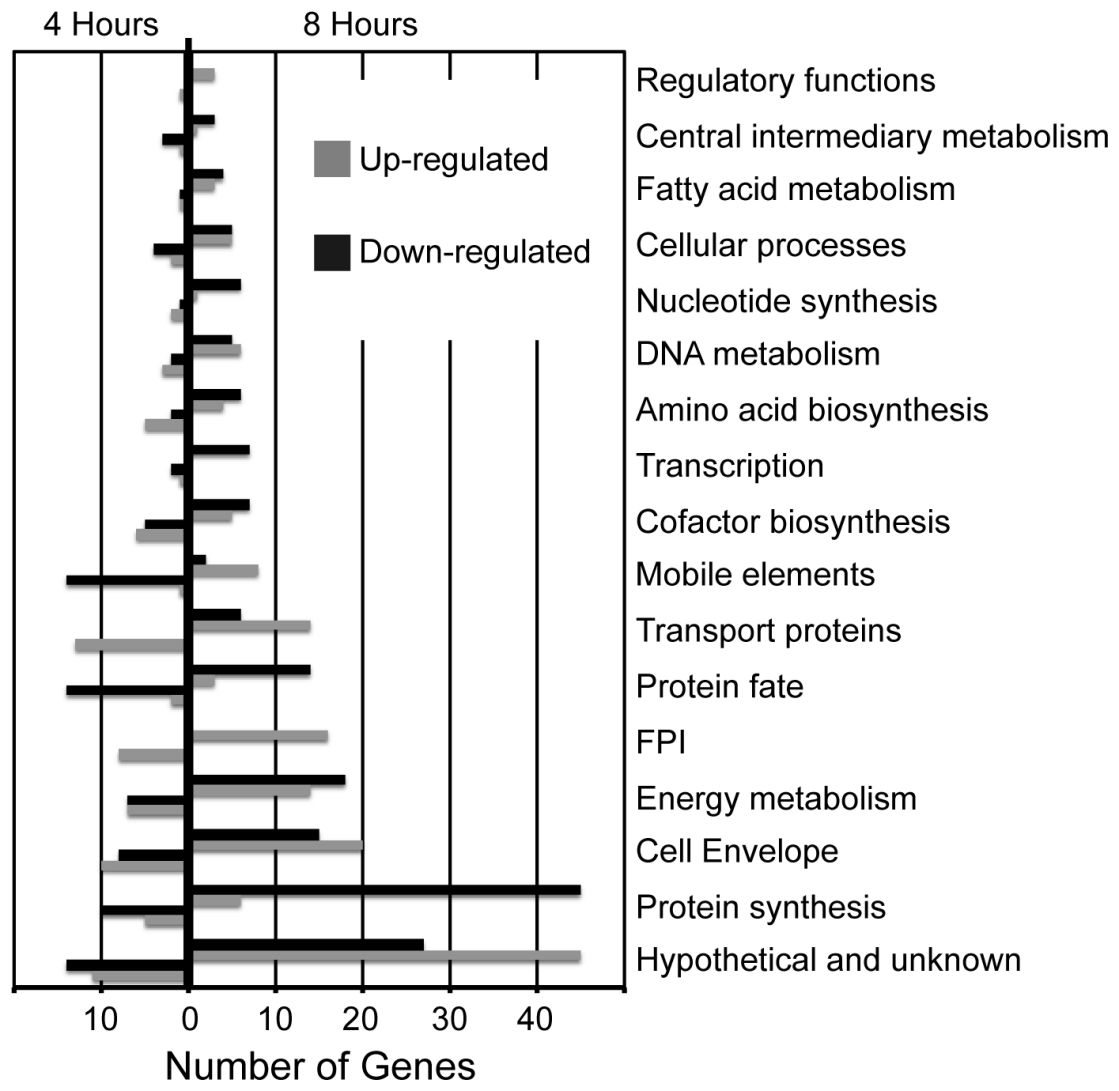
Number of genes up- and down-regulated, by functional category. All differentially expressed *F*. *tularensis* LVS genes were categorized by function, and the number of genes in each category were plotted according to whether their expression increased or decreased at 4 hours (left) and 8 hours (right) after infection.

To characterize the *F. tularensis* transcriptome dynamics associated with transition from growth in culture to infection of host cells, as well as the transition from phagosome to the cytosol, we performed a differential gene expression analysis of *F. tularensis* LVS before infection and after 4 or 8 hours of infection. Table S1 presents the biological duplicate FPKM (fragments per kilobase of transcript per million mapped reads) values and differential expression results for all *F. tularensis* genes, comparing the transcriptome of the culture grown inoculum to the transcriptome after 4 and 8 hours of infection. As expected, after 4 hours of infection we observed down-regulation of genes that are involved in protein synthesis, protein fate, and central intermediary metabolism (Figure 1); these results are consistent with slowing of replication during the transition from culture to infection. Genes that encode mobile elements, such as transposases, also showed down-regulated expression after 4 hours of infection. In contrast, genes encoding virulence determinants (e.g., transport proteins) and components of virulence-related biochemical pathways (e.g., biosynthesis of amino acids and cofactors) were up-regulated after 4 hours of infection. These up-regulated genes include many located within the Francisella pathogenicity island (FPI), which is known to play a critical role in *F. tularensis* virulence [23]. These trends in gene expression were still apparent after 8 hours of infection, with the exception of mobile element expression, which was up-regulated at the later time point. In both cases, and particularly at the later time point, many genes of unknown function showed differential expression. There are 21 differentially expressed genes that produce products of unknown function after 4 hours of infection and 60 genes after 8 hours (Tables S2 and S3, respectively). The highly up-regulated unknown genes at each time-point are especially interesting because they are likely to be important in specific stages of pathogenesis and yet have not been previously identified as virulence factors.

**Figure 2:**
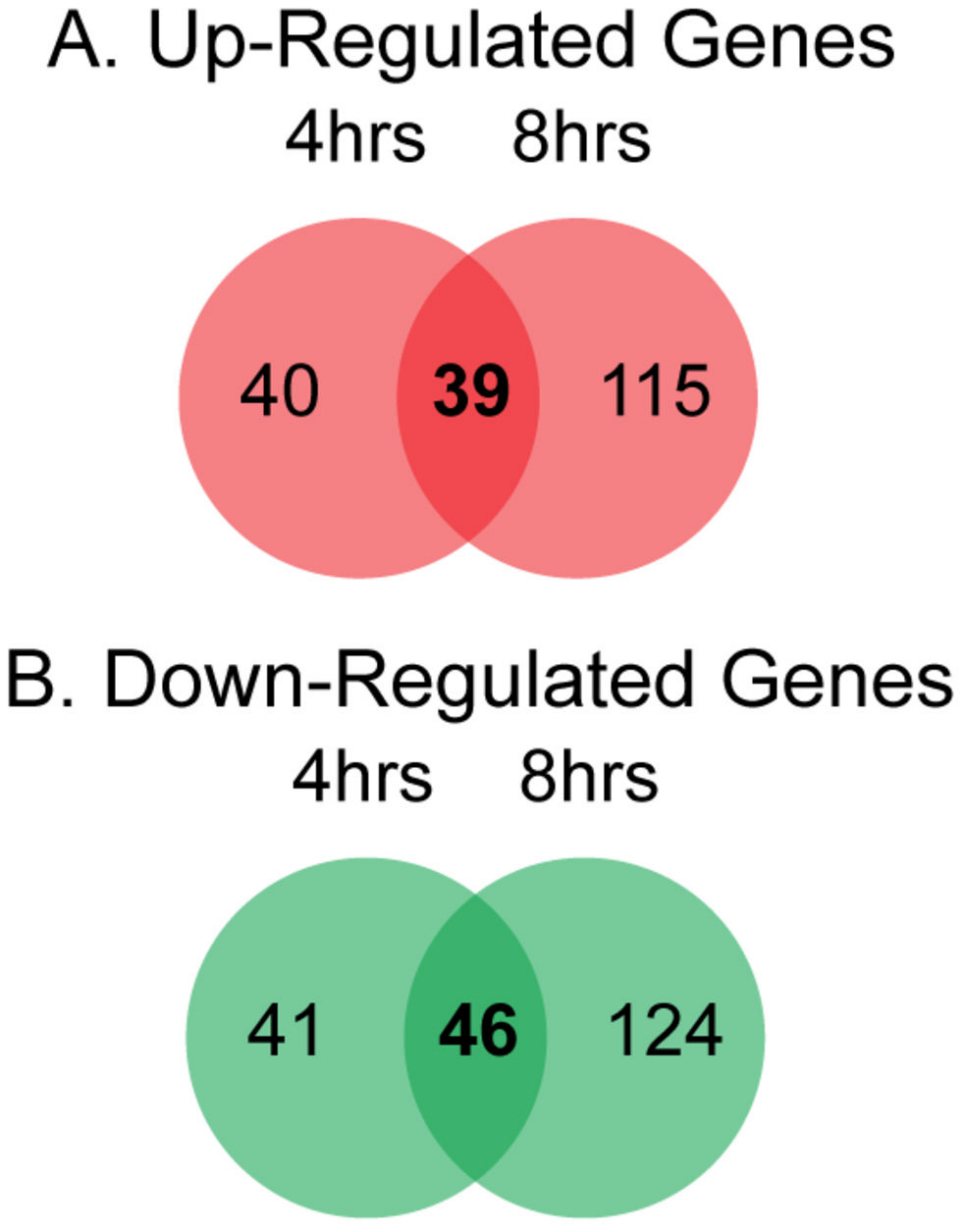
Comparison of the genes up- and down-regulated at each time point. The Venn diagrams depict the number of genes with significant changes in expression at both the 4 and 8-hour post-infection time points, with the number in the middle representing genes up- or down-regulated at both time points. A) Up-regulated genes. B) Down-regulated genes.

Table 1 lists the *F. tularensis* genes showing the largest changes in expression during transition from culture to infection. Strikingly, 30-40% of the most strongly up-regulated genes are of unknown function. Among the genes of known function, those that were most strongly up-regulated are involved in purine and amino acid biosynthesis, peptide transport, and competence. The genes most strongly down-regulated are involved in protein synthesis and central metabolism - functions predicted to play diminished roles during infection, as compared to exponential growth in culture. Interestingly, although the genes differentially expressed after 4 hours *versus* 8 hours of infection are closely related with respect to their annotated functions, only 39 (20.1%) of the up-regulated genes, and 46 (21.8%) of the down-regulated genes, were differentially expressed at both time points (Figure 2). The consistency with which these genes were differentially expressed suggests that they represent a core set of genes whose regulation is sensitive to the environmental changes associated with transition from culture to infection. Of the genes consistently up-regulated during infection (Table S4), ~20% are located within the FPI. Additionally, ~18% of these genes were categorized as transport and binding proteins, a group of proteins that include several genes implicated in virulence such as siderophore synthesis, and transmembrane peptide transport. Of the genes consistently down-regulated during infection (Table S5), ~49% are involved in protein synthesis and fate, and several others in biosynthesis of enzyme cofactors such as riboflavin, cyanophycin, and anthranilate. 

**Table 1:** Genes showing the largest changes in expression during infection.

**Gene ID**	**Name/Function**	**4hr Fold Change**	**Adj P-Value**	**Gene ID**	**Name/Function**	**8hr Fold Change**	**Adj P-Value**
FTL_0721	DedA family protein*	9.69	0.016	FTL_0815	PRC-barrel protein*	28.51	<0.001
FTL_1213	Unknown*	8.39	<0.001	FTL_1402	ISFtu1 transposase	13.91	<0.001
FTL_1216	Unknown*	8.16	<0.001	FTL_0953	methyltransferase	12.72	<0.001
FTL_1876	Outer membrane protein*	8.14	0.001	FTL_0814	PRC-barrel protein*	12.03	0.003
FTL_1509	Carboxypeptidase	7.82	0.007	FTL_0924	Oligopeptide transporter	10.90	0.037
FTL_0765	*vacJ*/lipoprotein	7.70	<0.001	FTL_1219	Aminotransferase	8.76	<0.001
FTL_0731	YhhQ family/purine regulon	6.69	0.011	FTL_1957	Heat shock	8.43	<0.001
FTL_0700	*comL*/competence	5.80	0.001	FTL_0816	Unknown*	8.18	.05
FTL_1219	Aminotransferase	5.42	<0.001	FTL_0123	Short chain dehydrogenase	8.13	<0.001
FTL_0691	Oligopeptide transport	5.24	<0.001	FTL_0473	Peptide deformylase	7.80	<0.001
FTL_1127	ISFtu1 transposase	-4.10	<0.001	FTL_0916	Ketol-acid reductoisomerase	-6.76	<0.001
FTL_0128	ISFtu1 transposase	-4.10	0.001	FTL_0243	*rplP*/ribosomal protein	-6.99	<0.001
FTL_0579	Nicotinate metabolism	-4.12	0.028	FTL_0204	Unknown*	-7.14	0.002
FTL_0732	Lactoylglutathione lyase	-4.17	0.001	FTL_0239	*rplB*/ribosomal protein	-7.25	<0.001
FTL_0227	Ribosome recycling factor	-4.31	0.002	FTL_0075	Riboflavin synthase	-7.35	<0.001
FTL_0266	ISFtu1 transposase	-4.81	0.028	FTL_1139	3-oxoacyl reductase	-7.52	<0.001
FTL_0799	Type IV pili lipoprotein*	-4.88	0.035	FTL_0241	*rplV*/ribosomal protein	-8.70	<0.001
FTL_0964	*hslU*/heat shock	-5.03	<0.001	FTL_1796	ATP synthase γ-subunit	-9.71	<0.001
FTL_0965	*hslV*/heat shock	-7.19	0.005	FTL_0244	L29 ribosomal protein	-11.24	<0.001
FTL_1128	Unknown*	-10.80	<0.001	FTL_0238	*rplW*/ribosomal protein	-11.49	<0.001

* Genes encoding products with unknown function

### Genomic localization of genes differentially expressed during infection

**Figure 3:**
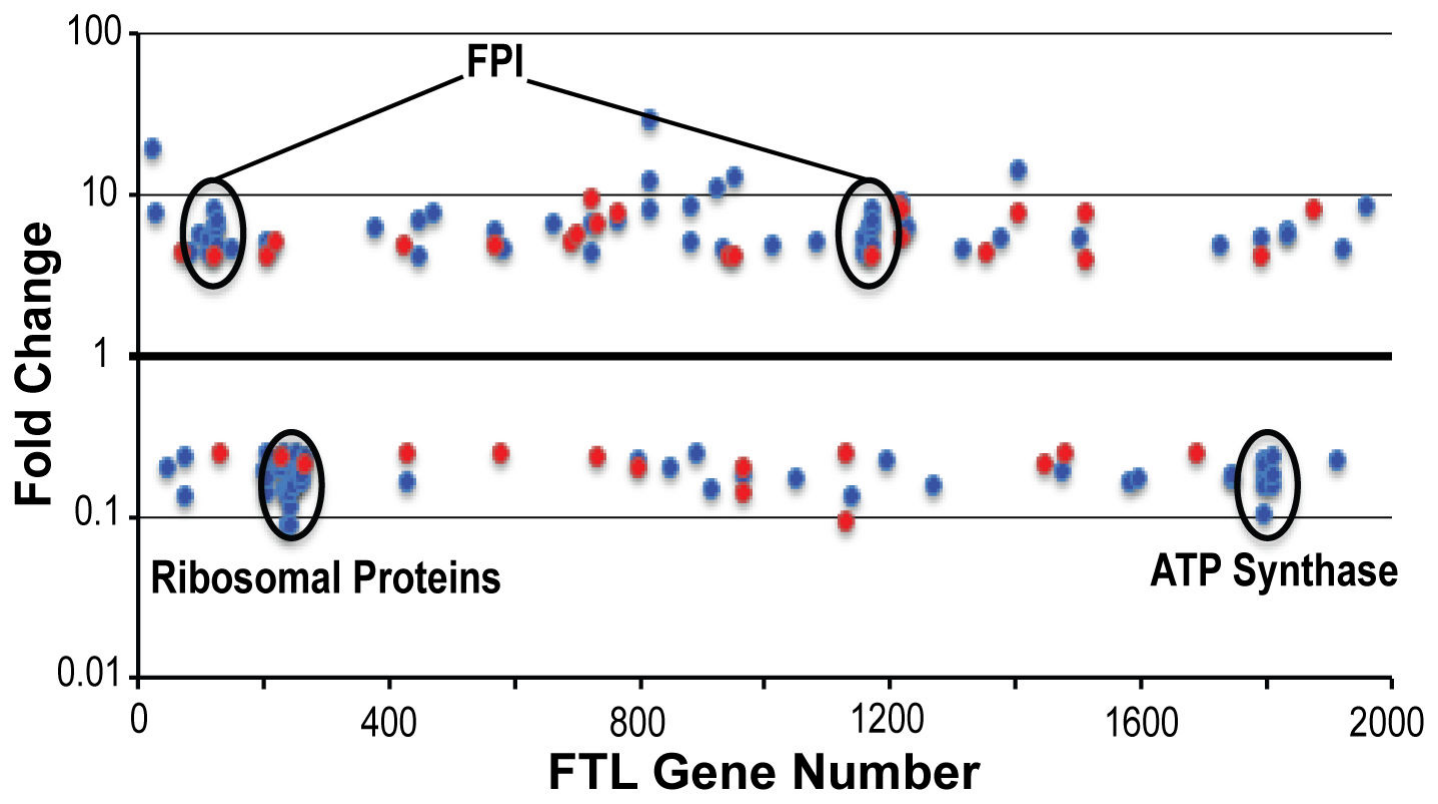
Differentially expressed genes plotted across the *F. tularensis* genome. All genes that had at least a 4-fold change in expression at either 4 hours (red) or 8 hours (blue) were plotted according to their gene ID number across the genome. The two copies of the FPI are highlighted in the up-regulated portion of the figure, and the ribosomal proteins and ATP synthase subunits are highlighted in the down-regulated portion of the figure.

To determine whether the genes differentially expressed during infection are located in particular regions of the *F. tularensis* genome, we identified the genes showing the largest changes in expression (> 4 fold) after 4 hours and 8 hours of infection, and mapped their locations within the genome. This analysis revealed that most of the differentially expressed genes are broadly distributed throughout the *F. tularensis* genome (Figure 3). However, notable exceptions include up-regulated genes mapping to the FPI (two copies in *F. tularensis* LVS); and two clusters of down-regulated genes, encoding ribosomal proteins and ATP synthase subunits, respectively. These results indicate that the *F. tularensis* genes most strongly induced or repressed during infection generally are not found within coordinately regulated gene clusters or chromosomal regions, with a few notable exceptions.

### Differential expression of transcriptional regulators

To gain further insight into the transcriptional shifts that occur during infection, we analyzed the expression of all known and putative *F. tularensis* LVS transcriptional regulators [24-31] (Table 2). After 4 hours of infection, the only transcriptional regulators up-regulated by at least 2-fold were *fevR* and FTL_1216. FevR has been shown to positively regulate expression of genes located within the FPI, as well as several other genes encoding virulence factors [32,33]. FTL_1216 is a putative transcriptional regulator that is conserved across many Gram-negative bacteria, but it has no known function or regulon. Given that FTL_1216 is the transcriptional regulator most strongly up-regulated after 4 hours of infection, but is not significantly up-regulated after 8 hours of infection, it seems likely that it plays an important role in phagosomal escape, but not in replication within the cytosol. The only transcriptional regulators significantly down-regulated after 4 hours of infection were *rpoD* and *migR*. Down-regulation of *rpoD*, which encodes the primary sigma factor, is consistent with the decrease in expression of numerous genes involved in aspects of cell growth (see Figure 1). On the other hand, down-regulation of *migR* would appear to contradict previous studies that demonstrated that MigR positively regulates *fevR* through the stress alarmone ppGpp [33,34]. This apparent contradiction may be explained by the high FPKM values of *mglA*, *sspA*, and *pmrA*, which also positively regulate *fevR* [32,34,35] and therefore may compensate for reduced expression of *migR*.

**Table 2:** Expression of known and putative transcriptional regulators.

**Gene ID**	**Name/Family**	**Control Expression (FPKM)**	**4hr Expression (FPKM**)	**DESeq Fold Change**	**Adj P-Value**	**8hr Expression (FPKM)**	**DESeq Fold Change**	**Adj P-Value**
FTL_0040	LysR family	2.19	1.82	1.06	1	0.21	0.17	0.793
FTL_0062	LysR famiy	1.56	1.19	1.89	1	0.40	1.38	1
FTL_0449	*fevR*	53.85	155.02	3.19*	<0.001	201.04	6.80*	<0.001
FTL_0552	*pmrA*	67.28	31.23	0.54	0.209	26.04	0.59	0.625
FTL_0662	*lexA*	21.49	9.16	0.62	0.763	11.84	1.06	1
FTL_0671	*panK1*	17.95	13.55	1.25	0.945	9.79	1.36	0.624
FTL_0689	AraC family	1.94	2.50	1.42	1	1.31	0.87	1
FTL_0742	LysR famliy	2.44	0.84	1.20	1	0.90	2.28	0.793
FTL_0780	Csp family	1.10	1.88	2.24	1	0.50	0.88	1
FTL_0844	LysR family	3.64	1.94	0.90	1	1.68	2.60	0.477
FTL_0851	*rpoH*	44.85	11.39	0.54	0.09	9.72	0.68	0.229
FTL_1014	*oxyR*	4.82	2.79	2.40	0.306	1.71	3.59*	0.004
FTL_1050	*rpoD*	37.00	10.44	0.38*	<0.001	8.08	0.44*	<0.001
FTL_1125	*hipA*	1.79	2.03	1.58	1	0.36	0.54	0.939
FTL_1126	XRE family	20.16	10.96	0.89	1	3.10	0.21	0.781
FTL_1176	LysR family	0	0	NA	1	0	NA	1
FTL_1185	*mglA*	45.19	53.93	1.86	0.062	10.63	0.42	0.088
FTL_1193	LysR family	1.00	0.49	0.82	0.823	0.64	0.86	1
FTL_1216	Unknown	3.50	18.8	8.11*	<0.001	2.01	2.38	0.391
FTL_1218	DGC	25.35	24.91	1.38	0.568	34.00	2.66*	<0.001
FTL_1231	*iscR*	12.83	14.33	2.08	0.458	18.78	3.21	0.088
FTL_1277	ROK famliy	7.97	4.48	0.80	1	2.12	0.71	0.838
FTL_1364	IclR family	66.38	25.62	0.55	0.085	24.03	0.67	0.21
FTL_1542	*migR*	26.86	5.49	0.29*	<0.001	3.77	0.32*	<0.001
FTL_1568	LysR family	0.76	0.40	1.22	1	0.16	1.26	1
FTL_1606	*sspA*	60.64	24.38	0.61	0.384	35.58	1.20	0.694
FTL_1634	LysR family	5.62	4.69	1.16	1	4.15	1.38	0.83
FTL_1665	*panK2*	0.70	1.18	2.34	0.713	1.00	2.65	0.371
FTL_1763	*qseC*	2.53	1.05	0.90	1	2.02	1.57	0.84
FTL_1831	*fur*	32.00	34.90	1.49	0.751	38.49	2.26*	0.037
FTL_1878	*kdpD*	5.27	4.05	1.1	1	3.37	1.68	0.296

* Differential expression (≥2 fold change in expression, p≤0.05)

We also observed up-regulation of *oxyR, fur*, and a diguanylate cyclase (DGC) (FTL_1218) after 8 hours of infection (Table 2). The *oxyR* and *fur* transcriptional regulators have been shown to promote formation of Fe-S clusters and mitigate oxidative damage in *E. coli* and many other bacteria [36-38] The gene product of *oxyR* was shown to be important in the response to oxidative stress in *F. novicida* during infection of Drosophila cells [39]. The ferric uptake regulator, or Fur, coordinates bacterial response to the iron-limited environment of the host, by promoting expression of the *fig*-*fsl* operon (siderophore production) [25,40,41] and other genes encoding virulence factors, including several located within the FPI [33]. Taken together, up-regulation of these three transcriptional regulators indicates that avoiding oxidative stress and scavenging iron and other limiting nutrients, is important for *F. tularensis* replication within the host cytoplasm. Diguanylate cyclase is responsible for the synthesis of cyclic di-GMP (cdGMP), a secondary messenger that promotes biofilm formation and inhibits virulence in *F. novicida* [42]. Expression of this gene during an infection might partially explain the decreased virulence of this Type B strain compared to more virulent Type A strains. Interestingly, all of the transcriptional regulators that were down-regulated after 8 hours of infection were also down-regulated at 4 hours. This suggests that despite their localization to different intracellular compartments at these two time points, the bacteria rely upon similar mechanisms to survive both stages of infection.

### Up-regulation of genes within the FPI

**Figure 4:**
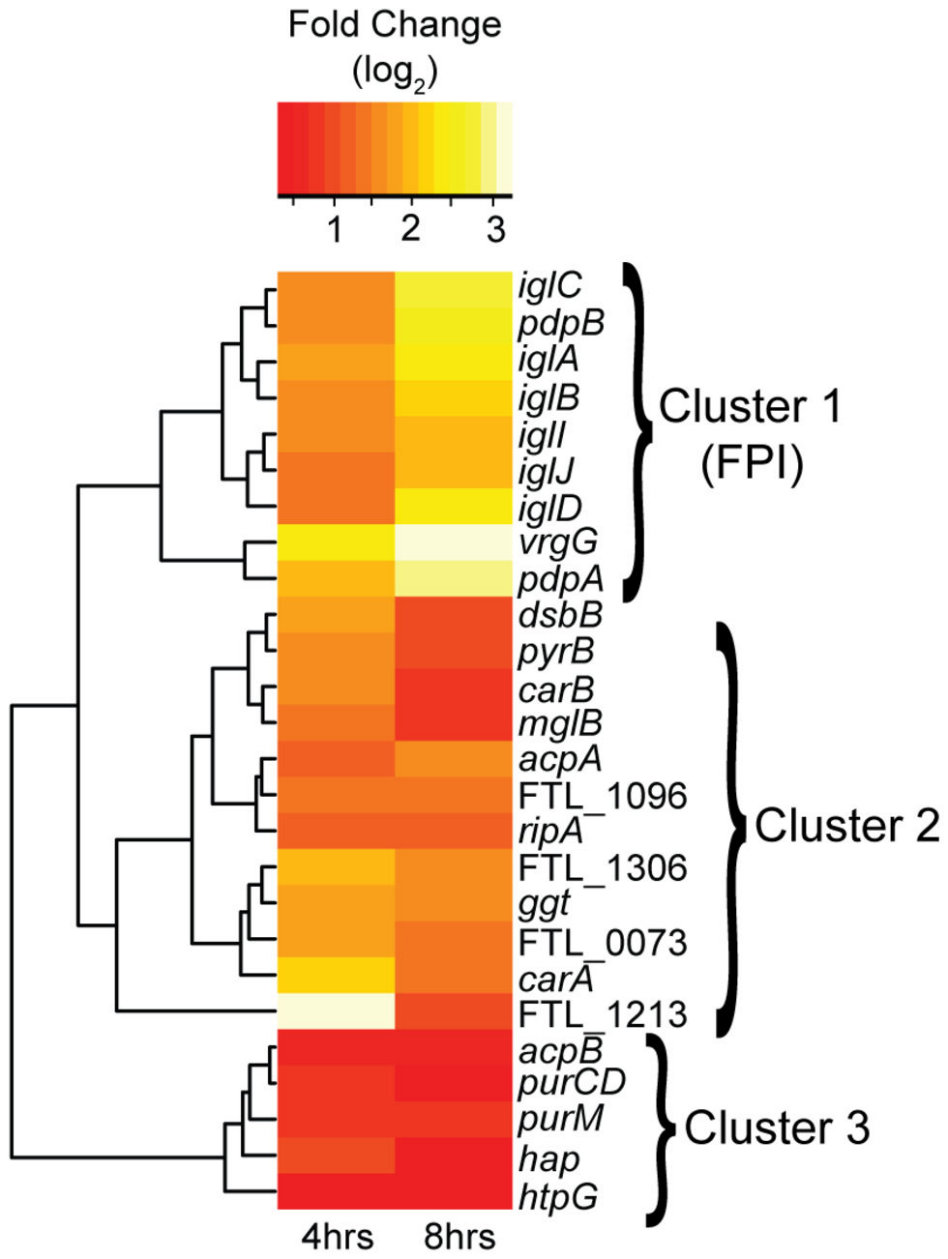
Heat map of virulence factor genes up- and down-regulated at each time point. Change in expression was determined for previously identified *F*. *tularensis* virulence factor genes at both post-infection time points, and then clustered to identify genes that are coordinately regulated. The cluster analysis segregated the genes into three groups. Cluster 1, in which the genes are up-regulated at both post-infection time points, is comprised entirely of genes in the FPI.

The FPI is an ~30 kb region encompassing 18 genes, that primarily encode an atypical type VI secretion system (T6SS) [43,44]. Most of the genes within the FPI have been implicated in virulence during at least one stage of infection, and were among the first recognized *Francisella* virulence factors [23,45]. We compared their expression levels during growth in culture *versus* after 4 hours or 8 hours of infection (Table 3). Consistent with our observation that *fevR*, a key positive regulator of FPI gene expression [32,33,46], is strongly up-regulated during infection, we found that the FPI genes were up-regulated after 4 hours of infection. Roughly half of the FPI genes show significant increases in expression at this stage of infection, and all except *pdpC* show higher levels of expression than measured in the bacteria grown in culture. After 8 hours of infection, all FPI genes were significantly up-regulated except for *anmK* and *pdpD*, both of which showed extremely low expression levels throughout the entire course of the experiment. Interestingly, *pdpE*, the only gene in the FPI that has not been shown to be necessary for full virulence [23], was significantly up-regulated after 8 hours of infection, indicating that while it may not be required for virulence, *pdpE* likely plays a role in *F. tularensis* replication in the cytoplasm. Also consistent with the FPI being coordinately regulated is the fact that the recognized virulence factors within the FPI all clustered with regard to their transcriptional shifts at 4 and 8 hours post-infection, showing the general trend of slight up-regulation at 4 hours followed by strong up-regulation at 8 hours (Figure 4). Overall, these results are consistent with those from previous studies that implicate the FPI and its encoded T6SS in *F. tularensis* virulence, and reinforce the idea that FevR regulates these genes.

**Table 3:** Expression of Francisella pathogenicity island (FPI) genes

**Gene ID**	**Name**	**Control Expression (FPKM)**	**4hr Expression (FPKM)**	**DESeq Fold Change**	**Adj P-Value**	**8hr Expression (FPKM)**	**DESeq Fold Change**	**Adj P-Value**
FTL_0109	*anmK*	2.30	1.08	1.52	1	1.21	1.63	1
FTL_0110	*pdpD*	0	0	NA	1	0	NA	1
FTL_0111	*iglA*	80.43	114.70	2.52*	<0.001	162.89	4.28*	<0.001
FTL_0112	*iglB*	87.71	127.90	2.14*	<0.001	138.17	3.74*	<0.001
FTL_0113	*iglC*	369.21	452.55	1.97	<0.001	908.81	5.43*	<0.001
FTL_0114	*iglD*	39.91	37.32	1.69	0.168	47.34	3.95*	<0.001
FTL_0115	*pdpE*	14.45	8.98	1.53	0.803	13.14	2.43*	0.025
FTL_0116	*pdpC*	11.10	7.34	0.94	1	11.27	2.21*	<0.001
FTL_0117	*iglJ*	14.78	15.49	1.73	0.347	13.44	3.01*	<0.001
FTL_0118	*iglI*	16.90	24.46	2.03*	0.047	30.40	2.96*	<0.001
FTL_0119	*dotU*	23.43	36.80	2.24	0.093	32.17	2.88*	0.002
FTL_0120	*iglH*	10.12	18.47	2.53*	0.004	22.44	4.40*	<0.001
FTL_0121	*iglG*	31.14	61.00	2.99*	0.033	88.83	6.12*	<0.001
FTL_0122	*iglF*	3.47	5.13	2.37	0.489	10.13	6.29*	<0.001
FTL_0123	*vrgG*	5.29	16.76	4.27*	0.047	15.51	8.13*	<0.001
FTL_0124	*iglE*	8.79	8.95	2.07	0.705	16.09	6.23*	<0.001
FTL_0125	*pdpB*	7.87	10.48	1.91	0.016	19.24	4.93*	<0.001
FTL_0126	*pdpA*	10.06	22.51	3.17*	<0.001	34.34	6.85*	<0.001

* Differential expression (≥2 fold change in expression, p≤0.05)

### Expression of non-FPI genes encoding virulence factors

While the FPI genes encode the best characterized of *F. tularensis* virulence factors, numerous non-FPI genes have been shown to be required for full virulence *in vitro* and *in vivo*, through use of mutational analysis screens [47-55]. However, mutational analysis has provided limited insight into the role of each virulence factor in the different stages of infection. For example, mutant bacteria that fail to enter the host cell or escape the phagosome are attenuated for overall virulence, yet they might be capable of intracellular growth and replication. To further elucidate the roles of non-FPI genes previously shown to be required for full virulence, we analyzed their expression during phagosomal escape and intracellular replication and compared it to their expression during growth in culture (Table 4). We found that five of the non-FPI genes encoding virulence factors were significantly up-regulated after 4 hours of infection, but only one of these was also significantly up-regulated after 8 hours of infection. Figure 4 shows two gene clusters (Cluster 2 and 3) that differ in expression from the pattern seen in the FPI. Cluster 2 contains genes that are up-regulated after four hours of infection, and show reduced expression after 8 hours of infection. Cluster 3 is comprised of the few virulence factors that are down-regulated at both time points relative to the bacteria in culture, suggesting that they may not be important for infection under the particular conditions of our model system, or that they play a role at earlier or later stages of infection not analyzed in this study [10].

**Table 4:** Expression of non-FPI genes encoding virulence factors.

**Gene ID**	**Name**	**Control Expression (FPKM)**	**4hr Expression (FPKM)**	**DESeq Fold Change**	**Adj P-Value**	**8hr Expression (FPKM)**	**DESeq Fold Change**	**Adj P-Value**
FTL_0028	*pyrB*	1.81	2.89	2.01	0.879	1.24	1.00	1
FTL_0029	*carB*	4.97	6.32	1.86	0.118	1.28	0.64	0.461
FTL_0030	*carA*	4.86	12.12	3.71*	0.007	2.92	1.67	0.576
FTL_0031	Hap	3.92	2.85	0.98	1	0.63	0.30	0.415
FTL_0073	Lipoprotein	23.98	40.22	2.44*	<0.001	19.02	1.57	0.161
FTL_0158	*acpA*	7.98	5.50	1.36	0.818	6.07	1.83	0.183
FTL_0267	*htpG*	135.54	30.60	0.32*	<0.001	16.20	0.23*	<0.001
FTL_0395	*purM*	31.28	18.23	0.81	0.922	10.96	0.73	0.489
FTL_0396	*purCD*	29.41	13.04	0.63	0.066	6.11	0.40*	<0.001
FTL_0552	*pmrA*	67.28	31.23	0.55	0.209	26.04	0.83	0.625
FTL_0766	*ggt*	4.38	8.06	2.59*	0.04	4.00	1.93	0.182
FTL_0889	*acpC*	0	0	NA	1	0	NA	1
FTL_1096	Thioredoxin	26.23	27.47	1.49	0.289	20.31	1.54	0.147
FTL_1184	*mglB*	31.23	24.99	1.60	0.971	9.16	0.75	0.98
FTL_1213	Unknown	2.67	16.30	8.39*	<0.001	1.09	0.99	1
FTL_1306	Unknown	13.50	29.18	2.99*	<0.001	14.13	2.10*	0.029
FTL_1670	*dsbB*	9.39	14.51	2.25	0.522	4.12	0.95	1
FTL_1732	*acpB*	11.70	5.21	0.59	0.868	0.98	0.46	0.437
FTL_1914	*ripA*	167.89	138.03	1.20	0.608	126.99	1.42	0.095

* Differential expression (≥2 fold change in expression, p≤0.05)

The non-FPI genes encoding virulence factors that were up-regulated after 4 hours of infection are *carA*, *ggt*, FTL_0073, FTL_1213, and FTL_1306. Mutants of *carA* are uracil auxotrophs that are unable to escape the phagosome in neutrophils [52], consistent with the idea that CarA plays an important role in phagosomal escape. ϒ-glutamyl transpeptidase (GGT) was shown to allow *F. tularensis* to acquire cysteine during replication within the host cell cytosol [47]; a *ggt* deletion mutant in the *F. tularensis* SCHU S4 background has even been proposed as a potential vaccine candidate [56]. FTL_0073 (FTT_1676 in SCHU S4) encodes a lipoprotein that has been shown to be required for both phagosomal escape and intracellular growth [12]. FTL_1213 (FTT_0989), which is thought to encode a secreted transglutaminase, was shown by Brotcke and colleagues to be required for full virulence in cultured macrophages as well as in mice [46]; however, Wehrly and colleagues found no such requirement when infecting cultured macrophages [12]. The fact that we observed high levels of FTL_1213 expression after 4 hours of infection, and reduced levels of expression after 8 hours of infection, suggests that its gene product may be more important for phagosomal escape than for subsequent stages of infection. FTL_1306 (FTT_0369c), the only non-FPI virulence-associated gene that was significantly up-regulated at both time points, has been shown to be required for *F. tularensis* replication in the cytosol [12]; however, its specific function has yet to be determined.

It should be noted that two non-FPI genes encoding virulence factors (*htpG* and *purCD*) were significantly down-regulated after 4 hours and 8 hours of infection. HtpG is a heat shock protein that was shown to be required for virulence in macrophages and mice [54,55], whereas *purCD* was shown to be required for purine biosynthesis during infection [57,58]. It remains to be determined whether down-regulation of *htpG* and *purCD* during infection is observed in other model systems, or is a unique feature of *F. tularensis* infection of P388D1 macrophages.

In summary, the results of these analyses, as well as those presented in Figure 4, fit well with those from previous studies, and show that expression of the non-FPI virulence-associated genes is less coordinated than that of the FPI genes. Further research will be necessary to elucidate the specific functions and roles in pathogenesis of the uncharacterized but up-regulated virulence factors FTL_0073, FTL_1213, and FTL_1306.

## Discussion

*F. tularensis* has the ability to infect multiple cell types and exist within multiple intracellular compartments during infection of the host. The requirements for survival and proliferation during infection have been studied both *in vitro* and *in vivo*, primarily through mutational analysis to identify the genes that are critical for virulence [47-55]. While this approach has been highly successful in discovering genes that are required for full virulence of the bacteria in a given model system of infection, it is not without its disadvantages. One significant drawback to these types of studies is that they often fail to determine the stage(s) of infection for which the genes are required. For example, a gene that is required for the initial entry into a host cell will be identified as critical for virulence, however it is typically not possible to determine whether this gene is also involved in phagosomal escape or replication within the host cell cytosol, as a mutant for that gene will not proceed to those stages of infection. To understand when and where genes are expressed throughout the course of an infection, transcriptional analyses are required. Global analysis of the transcriptome can be performed using either microarrays designed specifically for the pathogen of interest or, more recently, by sequencing total RNA (RNA-Seq) from an infected sample. While RNA-Seq is a relatively new technology, its sensitivity, dynamic range, low cost, and ability to detect non-protein-coding transcripts is unmatched by microarray-based approaches. 

A major consideration for either transcriptomics approach is that the RNA recovered from virtually any infection is primarily host-derived, with the pathogen RNA outnumbered by well over 100-fold [14,59]. Using RNA-Seq to analyze the pathogen transcriptome under these circumstances becomes expensive, as deeper sequencing is required to get enough reads for a gene-level analysis throughout the course of an infection. Prior work in our lab has demonstrated the effectiveness of a capture-based approach to enrich for pathogen transcripts from infected cells [14]. This technique relies on the use of biotinylated probe sequences randomly generated from the entire bacterial genome, ensuring that all possible transcripts can be captured. Double-stranded and tagged cDNA generated from the infected sample [60] are mixed with a large excess of capture probe. The mixture is denatured, and stringent hybridization conditions are established to allow the pathogen-derived cDNA to anneal to complementary capture probe sequences. The hybridization mixture is then adsorbed to a monomeric avidin column, washed repeatedly to remove cDNA non-specifically bound to the probe, and the remaining cDNA released from the column to generate a pool enriched for pathogen-derived sequences. The short tags at the ends of the cDNA allow PCR-mediated addition of full-length sequencing adaptors, whereas the probes lack these tags preventing inadvertent sequencing of the probe. Using a *F. tularensis* LVS infection model, we previously demonstrated unbiased enrichment of bacterial transcripts by upwards of 50-fold [14]. This enrichment of pathogen transcripts allows for much more efficient sequencing of the bacterial transcriptome at any stage of infection, as compared to brute-force RNA-Seq without enrichment.

Given the proven ability of our pathogen capture approach to enrich for *F. tularensis* transcripts present in infected samples, we employed the technique to perform a differential gene expression analysis comparing the transcriptomes of the bacteria before and after infection. By observing the transcriptional profiles of the bacteria at two distinct time points after entry into the host cell, we hypothesized that it would be possible to determine sets of genes that were important in two critical stages of the infection: phagosomal escape, and cytosolic growth. At each time point we analyzed the global transcriptional shifts with respect to changes in expression of functional categories of genes as well as sets of known and putative transcriptional regulators and virulence factors.

The challenge faced by bacteria as they shift from culture to infection of host cells can be summed up as a change from replication in a protected environment to survival in a threatening environment. Consistent with this idea, we found that transition from culture to infection was generally associated with up-regulation of genes involved in virulence and stress response, and down-regulation of genes involved in replication. These results were consistent with expectations, and indicated that our techniques are effective at detecting the previously-identified transcriptional shifts that occur during infection. However, the switch from growth in culture to infection of host cells is both spatially and temporally dynamic, with different cell types and intracellular compartments presenting different challenges to bacterial survival. This is why, when the two stages of infection were analyzed in greater detail, we observed analogous but distinctive gene expression patterns associated with phagosomal escape and cytosolic replication. 

Perhaps the greatest insight into the global transcriptional shifts that occur during different stages of infection can be obtained through analysis of transcriptional regulators. Interestingly, very few regulatory proteins have been identified in *F. tularensis*; indeed, its genome shows a complete lack of classically arranged two-component regulatory systems and only one alternative sigma factor [24,35,61,62]. Previous work has identified only 31 potential transcriptional regulators in *F. tularensis* LVS [24-30], a number that is considerably lower than in related bacteria such as *E. coli*, which has over 250 putative transcription factors [63]. Despite the substantial number of genes that showed significant changes in expression during the course of the infection (405 in total), we found that only 7 of the previously identified regulators showed a significant change in expression relative to the bacteria grown in culture. Combined with the fact that there are so few transcription factors identified to date, this may indicate that *F. tularensis* regulates gene expression using different mechanisms than those examined in this study. These could include use of unusual transcription factors, anti-sense RNAs, or post-transcriptional modifications that lead to the transcriptome shifts that we observed. One of the more interesting observations in this work is the up-regulation of the putative transcription factor FTL_1216 after 4 hours of infection. Though its function is not yet known, the gene is conserved in many bacteria and, based on our results, likely plays a role in regulating the expression of genes involved in phagosomal escape. More work will be necessary to determine the exact nature of its role in virulence and to discover the genes in its regulon.

Like all pathogens, *F. tularensis* expresses a suite of virulence factors required for completion of its pathogenic life cycle. The best characterized of these are the genes comprising the FPI and its encoded T6SS. While there is still much work to be done to understand the precise functions of these genes, it is clear from our study, and from those of others, that FPI genes play important roles in phagosomal escape and cytosolic replication. Indeed, the FPI genes were the only virulence-associated genes (aside from FTL_1306) that showed up-regulation after both 4 hours and 8 hours of infection. A number of non-FPI genes are associated with virulence, but strikingly few were differentially expressed during infection in our model system. One intriguing exception is FTL_1213, which showed up-regulation after 4 hours of infection, suggesting that this virulence-associated gene may play an important role in phagosomal escape. Its chromosomal proximity and similar expression profile to FTL_1216, a putative transcriptional regulator, suggests that FTL_1213 may belong to the regulon controlled by FTL_1216, perhaps acting in concert with other similarly regulated virulence factors to promote escape from the phagosome. 

In this study we also observed several genes that were strongly up-regulated during infection, and yet have not been previously identified as virulence factors. Genes such as FTL_0721 and FTL_1876 are of particular note, as they are strongly up-regulated early in infection, and the fact that their protein products are predicted to include transmembrane domains suggests that they may localize to the bacterial cell surface. Although the precise functions of these genes are not yet understood, they may make attractive therapeutic targets at early stages of tularemia. We also identified 39 genes encoding proteins of unknown function that showed significant changes in expression after 8 hours of infection (Table S3), and therefore likely play important roles during cytosolic replication. Together these uncharacterized up-regulated genes make for intriguing topics of future research projects, and indicate that there is still a significant amount to learn about the molecular mechanisms of *F. tularensis* pathogenesis.

## Materials and Methods

### Infection of Murine Macrophages and RNA Extraction

Infections and RNA extraction were performed in biological duplicates as previously described [14]. Briefly, P388D1 cells obtained from ATCC (ATCC^®^ CCL-46™) were grown in six-well microtiter plates overnight in RPMI media (Life Technologies) supplemented to a final concentration of 10% fetal bovine serum. *Francisella tularensis* spp. *holarctica* LVS obtained from BEI (NR-646) was grown in *Francisella* broth (BHI supplemented with 17.5g/L casamino acids and 2% isovitalex) overnight at 37°C in a shaking incubator. Bacterial concentrations were determined by OD_600_ with comparison to a previously established standard curve. Approximately 1.45x10^6^ P388D1 cells were infected with the overnight culture of *F. tularensis* LVS to produce a multiplicity of infection (MOI) of 10. Biological duplicate samples were taken at 4 hours and 8 hours post infection, washed twice with PBS to remove non-adherent or non-internalized bacteria, and 1mL of RNAzol (Molecular Research Center, Inc.) was immediately added to each well. RNA was extracted in combination with the Direct-zol kit (Zymo Research) according to the manufacturer’s instructions. Total RNA was also extracted in biological duplicates from 1mL of the overnight *F. tularensis* culture used as the inoculum, using the technique described above. In all cases, the RNA was quantitated using a Qubit (Life Technologies) and run on a BioAnalyzer (Agilent) to determine its integrity.

### RNA-Seq library preparation and sequencing

Double-stranded, tagged cDNA was generated from total RNA as previously described [14,60]. For the infection samples, 20ng of cDNA was mixed with 2μg of biotinylated probes generated against the entire *F. tularensis* genome using the BioPrime DNA Labeling System (Life Technologies), denatured, and allowed to hybridize overnight. Following hybridization, probe-bound *F. tularensis* transcripts were selectively removed from the pool using monomeric avidin agarose (Pierce/Thermo) as previously described [14]. The optimal cycle number for indexing PCR was determined by qPCR, and samples were barcoded using custom indexing primers [60]. Libraries were combined in equal molar amounts and visualized using the Bionalyzer (Agilent). The Vincent J. Coates Genomics Sequencing Laboratory (University of California, Berkeley) performed 100-base, single-end sequencing using an Illumina HiSeq 2000. All quality filtered reads have been deposited in the NCBI Sequence Read Archive (SRA) with the accession number PRJNA213748

### RNA-Seq data analysis and statistical determination of differentially expressed genes

Raw reads were processed using a previously described quality filter designed to remove low quality reads or sections of reads as well as any sequences derived from the sequencing adaptors or primers [64]. The quality filtered FASTQ files were mapped to the *Francisella tularensis* LVS genome (NC_007880) with Bowtie 2 in local alignment mode [65]. The alignments were converted and sorted with the SAMtools package [66]. For the differential expression analysis, read counts were generated for each CDS in the NCBI RefSeq annotation of the LVS genome with the BEDTools multicov tool [67]. Differentially expressed genes were identified at each time point with the R package DESeq [68], by comparing the read counts of each CDS at four and eight hours to those in the culture-grown control. This package tests for differential expression through the application of the negative binomial distribution and a shrinkage estimator for the distribution’s variance. Normalized expression levels among the various samples were obtained by estimating the total sequencing depths for each sample as the median of the ratios of the sample’s counts to geometric mean across all samples. Further details of the statistical analyses can be found in the DESeq vignette (http://www.bioconductor.org/ packages/2.12/bioc/vignettes/DESeq/inst/doc/DESeq.pdf). Genes were identified as differentially expressed when the DESeq calculated adjusted p-value was less than 0.05 and the change in expression was at least two-fold up or down. FPKM values for each annotated CDS were calculated from the alignments by providing Cufflinks with a reference annotation [69]. Each gene’s functional category was determined by the J. Craig Venter Institute’s Comprehensive Microbial Resource (cmr.jcvi.org)

## Supporting Information

Table S1 **FPKM values and differential expression results for all *F. tularensis* LVS genes in duplicate at each time point compared to the control.**
(XLSX)

Table S2 **Genes of unknown function that are differential expressed after 4 hours of infection.**
(DOC)

Table S3 **Genes of unknown function that are differential expressed after 8 hours of infection.**
(DOC)

Table S4 **Genes up-regulated at both the 4 and 8-hour time points.**
(DOC)

Table S5 **Genes down-regulated at both the 4 and 8-hour time points.**
(DOC)
