# Supplementary material for: Use of a Capture-Based Pathogen Transcript Enrichment Strategy for RNA-Seq Analysis of the Francisella Tularensis LVS Transcriptome during Infection of Murine Macrophages
Source: PLoS One. 2013 Oct 14;8(10):e77834. doi: 10.1371/journal.pone.0077834 (PMC3796476; doi:10.1371/journal.pone.0077834)
Supplement: Table S2 — Genes of unknown function that are differential expressed after 4 hours of infection. (DOC) [file pone.0077834.s002.doc]

**Table S2: Genes of unknown function that are differentially expressed after 4 hours of infection**

| **Gene ID** | **Control FPKM** | **4hrs FPKM** | **Fold Change** | **Adj P-Value** | **Gene Info** |
| --- | --- | --- | --- | --- | --- |
| FTL_0721 | 2.54 | 16.99 | 9.69 | 0.016 | DedA family protein |
| FTL_1213 | 2.67 | 16.30 | 8.39 | <0.001 | hypothetical protein |
| FTL_1216 | 3.49 | 18.80 | 8.16 | <0.001 | conserved hypothetical protein |
| FTL_1509 | 3.18 | 10.65 | 7.82 | 0.007 | carboxypeptidase |
| FTL_0731 | 2.45 | 8.35 | 6.69 | 0.011 | YhhQ family protein |
| FTL_0700 | 3.97 | 17.51 | 5.80 | 0.001 | conserved hypothetical lipoprotein |
| FTL_1219 | 24.76 | 96.48 | 5.42 | <0.001 | Aminotransferase, class II |
| FTL_0941 | 11.92 | 30.91 | 4.09 | 0.005 | hypothetical protein |
| FTL_0449 | 53.84 | 155.02 | 3.21 | <0.001 | hypothetical protein |
| FTL_1306 | 13.49 | 29.17 | 2.99 | <0.001 | hypothetical protein |
|  |  |  |  |  |  |
| FTL_1144 | 88.46 | 19.15 | -2.13 | 0.022 | conserved hypothetical protein |
| FTL_0929 | 54.64 | 15.83 | -2.33 | 0.02 | conserved hypothetical protein |
| FTL_0572 | 39.87 | 9.47 | -2.70 | <0.001 | conserved hypothetical protein |
| FTL_0417 | 27.33 | 6.49 | -2.94 | 0.03 | Transposase |
| FTL_0460 | 25.77 | 6.19 | -3.03 | 0.04 | hypothetical protein |
| FTL_1293 | 70.50 | 15.90 | -3.13 | 0.018 | hypothetical protein |
| FTL_0905 | 89.38 | 17.33 | -3.33 | <0.001 | low molecular weight phosphotyrosine protein phosphatase |
| FTL_1542 | 26.86 | 5.49 | -3.33 | <0.001 | conserved hypothetical protein |
| FTL_0867 | 18.24 | 3.51 | -3.85 | 0.002 | hypothetical protein |
| FTL_0799 | 29.51 | 3.50 | -4.76 | 0.036 | Type IV pili lipoprotein. |
| FTL_1128 | 19.81 | 2.83 | -11.11 | <0.001 | hypothetical protein |
