# Supplementary material for: Use of a Capture-Based Pathogen Transcript Enrichment Strategy for RNA-Seq Analysis of the Francisella Tularensis LVS Transcriptome during Infection of Murine Macrophages
Source: PLoS One. 2013 Oct 14;8(10):e77834. doi: 10.1371/journal.pone.0077834 (PMC3796476; doi:10.1371/journal.pone.0077834)
Supplement: Table S3 — Genes of unknown function that are differential expressed after 8 hours of infection. (DOC) [file pone.0077834.s003.doc]

**Table S3: Genes of unknown function that are differentially expressed after 8 hours of infection**

| **Gene ID** | **Control FPKM** | **8hrs FPKM** | **Fold Change** | **Adj P-Value** | **Gene Info** |
| --- | --- | --- | --- | --- | --- |
| FTL_0815 | 4.35 | 38.90 | 28.51 | <0.001 | PRC-barrel |
| FTL_0814 | 2.43 | 3.66 | 12.03 | 0.003 | conserved hypothetical protein |
| FTL_1219 | 24.76 | 110.06 | 8.76 | <0.001 | Aminotransferase, class II |
| FTL_0816 | 29.00 | 46.12 | 8.18 | 0.05 | hypothetical protein |
| FTL_0767 | 2.87 | 9.06 | 6.89 | <0.001 | conserved hypothetical protein |
| FTL_0449 | 53.84 | 201.04 | 6.87 | <0.001 | hypothetical protein |
| FTL_0663 | 5.25 | 24.37 | 6.75 | <0.001 | hypothetical protein |
| FTL_1223 | 19.93 | 70.76 | 6.54 | <0.001 | hypothetical protein |
| FTL_0097 | 35.38 | 96.87 | 5.80 | <0.001 | hypothetical protein |
| FTL_0880 | 1.96 | 5.55 | 5.05 | 0.009 | hypothetical protein |
| FTL_0147 | 3.63 | 8.64 | 4.67 | <0.001 | hypothetical protein |
| FTL_1317 | 26.48 | 63.26 | 4.66 | <0.001 | hypothetical protein |
| FTL_0935 | 16.49 | 36.23 | 4.52 | 0.026 | conserved hypothetical protein |
| FTL_1217 | 26.29 | 40.29 | 3.94 | <0.001 | hypothetical protein |
| FTL_0723 | 70.36 | 137.64 | 3.84 | <0.001 | hypothetical protein |
| FTL_1494 | 25.71 | 53.57 | 3.77 | <0.001 | hypothetical protein |
| FTL_0937 | 5 | 8.52 | 3.53 | 0.007 | hypothetical protein |
| FTL_0582 | 18.08 | 33.17 | 3.52 | <0.001 | conserved hypothetical protein |
| FTL_1892 | 32.14 | 57.76 | 3.44 | <0.001 | hypothetical protein |
| FTL_0834 | 14.98 | 24.73 | 3.43 | 0.002 | Rhodanese-like family protein |
| FTL_0465 | 3.2 | 5.82 | 3.38 | 0.028 | BNR/Asp-box repeat protein |
| FTL_0511 | 11.01 | 18.56 | 3.33 | 0.002 | hypothetical protein |
| FTL_0637 | 16.33 | 18.6 | 3.31 | <0.001 | conservered hypothetical protein |
| FTL_0846 | 11.85 | 14.8 | 3.30 | 0.041 | isochorismatase hydrolase family protein |
| FTL_1753 | 11.3 | 13.78 | 3.19 | 0.017 | ion channel protein, fragment |
| FTL_0901 | 8.7 | 8.78 | 3.19 | 0.01 | monooxygenase family protein |
| FTL_0722 | 7.19 | 8.15 | 2.93 | 0.05 | DedA family protein |
| FTL_0755 | 282.35 | 252.28 | 2.93 | <0.001 | hypothetical protein |
| FTL_0902 | 26.4 | 130.52 | 2.92 | 0.019 | oxidoreductase |
| FTL_0265 | 82.08 | 122.3 | 2.87 | <0.001 | hypothetical protein |
| FTL_0995 | 12.24 | 15.53 | 2.75 | 0.019 | haloacid dehalogenase |
| FTL_1218 | 25.35 | 34 | 2.69 | <0.001 | hypothetical protein |
| FTL_0411 | 20.58 | 24.8 | 2.47 | 0.002 | hypothetical protein |
| FTL_1088 | 50.7 | 52.77 | 2.46 | 0.006 | hypothetical protein |
| FTL_0222 | 14.27 | 17.63 | 2.37 | 0.004 | conserved hypothetical protein |
| FTL_1756 | 20.09 | 23.11 | 2.34 | <0.001 | anaerobic glycerol-3-phosphate dehydrogenase |
| FTL_1075 | 35.87 | 40.04 | 2.31 | 0.002 | hypothetical protein |
| FTL_0457 | 1036.08 | 862.52 | 2.25 | <0.001 | cold shock protein |
| FTL_1306 | 13.49 | 14.13 | 2.10 | 0.029 | hypothetical protein |
|  |  |  |  |  |  |
| FTL_0928 | 45.17 | 10.34 | -2.22 | 0.071 | DJ-1/PfpI family protein |
| FTL_1427 | 13.43 | 1.98 | -2.38 | 0.048 | conserved hypothetical protein |
| FTL_0905 | 89.38 | 19.15 | -2.44 | 0.003 | low molecular weight (LMW) phosphotyrosine protein phosphatase |
| FTL_0602 | 45.41 | 9.65 | -2.44 | 0.007 | formyl transferase |
| FTL_1811 | 78.69 | 13.65 | -2.50 | 0.008 | conserved hypothetical protein |
| FTL_0590 | 5.36 | 1.02 | -2.78 | 0.005 | ATp-dependent helicase |
| FTL_0929 | 54.64 | 7.41 | -2.86 | <0.001 | conserved hypothetical protein |
| FTL_0211 | 16.55 | 3.03 | -2.86 | 0.037 | hypothetical protein |
| FTL_1332 | 48.96 | 6.6 | -2.94 | 0.043 | hesB family protein |
| FTL_1542 | 26.86 | 3.77 | -3.13 | <0.001 | conserved hypothetical protein |
| FTL_1371 | 22.15 | 3.28 | -3.13 | 0.01 | hypothetical protein |
| FTL_0906 | 48.4 | 5.83 | -3.57 | <0.001 | ATP/GTP-binding protein |
| FTL_0212 | 9.08 | 0.87 | -3.85 | 0.048 | hypothetical protein |
| FTL_1128 | 19.81 | 3.04 | -3.85 | 0.003 | hypothetical protein |
| FTL_0203 | 13.91 | 1.43 | -4.00 | 0.023 | hypothetical membrane protein |
| FTL_0799 | 29.51 | 3.23 | -4.55 | 0.006 | Type IV pili lipoprotein. |
| FTL_0044 | 3.49 | 0.36 | -5.00 | 0.01 | Transglutaminase-like superfamily domain protein |
| FTL_0205 | 14.06 | 0.92 | -5.88 | 0.018 | TPR repeat |
| FTL_1048 | 126.46 | 11.42 | -5.88 | <0.001 | conserved hypothetical protein |
| FTL_1579 | 128.2 | 10.06 | -5.88 | <0.001 | hypothetical protein |
| FTL_0204 | 12.24 | 0.32 | -7.14 | 0.002 | TPR (tetratricopeptide repeat) domain protein |
