# Supplementary material for: Use of a Capture-Based Pathogen Transcript Enrichment Strategy for RNA-Seq Analysis of the Francisella Tularensis LVS Transcriptome during Infection of Murine Macrophages
Source: PLoS One. 2013 Oct 14;8(10):e77834. doi: 10.1371/journal.pone.0077834 (PMC3796476; doi:10.1371/journal.pone.0077834)
Supplement: Table S4 — Genes up-regulated at both the 4 and 8-hour time points. (DOC) [file pone.0077834.s004.doc]

| **Gene ID** | **Name/Function** | **Category** |
| --- | --- | --- |
| FTL_0070 | *rpsT* | Protein synthesis |
| FTL_0099 | Tryptophan synthase – beta subunit | Amino acid biosynthesis |
| FTL_0111 | *iglA* | FPI |
| FTL_0112 | *iglB* | FPI |
| FTL_0118 | *iglI* | FPI |
| FTL_0119 | *dotU* | FPI |
| FTL_0120 | *iglH* | FPI |
| FTL_0121 | *iglG* | FPI |
| FTL_0123 | *vrgG* | FPI |
| FTL_0126 | *pdpA* | FPI |
| FTL_0207 | Pyrrolidone-caroxylate peptidase | Protein Fate |
| FTL_0208 | Cytochrome C-type biogenesis protein | Cell envelope |
| FTL_0209 | DNA polymerase III – chi subunit | DNA metabolism |
| FTL_0221 | Amino acid permease | Transport and binding proteins |
| FTL_0425 | Type IV pili glycosylation protein | Cell envelope |
| FTL_0449 | Unknown | Hypothetical and unknown |
| FTL_0473 | Peptide deformylase | Protein Fate |
| FTL_0491 | Outer membrane lipoprotein | Energy metabolism |
| FTL_0569 | Hypothetical OMP | Cell envelope |
| FTL_0691 | H+ dependent oligopeptide transport | Transport and binding proteins |
| FTL_0700 | Lipoprotein | Hypothetical and unknown |
| FTL_0721 | DedA family protein | Hypothetical and unknown |
| FTL_0806 | Amino acid transporter | Transport and binding proteins |
| FTL_0807 | Major facilitator transporter | Transport and binding proteins |
| FTL_0895 | Histone-like protein HU | DNA metabolism |
| FTL_0898 | Host-factor I for bacteriophage Q | Regulatory functions |
| FTL_0953 | Methyltransferase | Biosynthesis of cofactors, prosthetic groups, and carriers |
| FTL_1219 | Hypothetical aminotransferase | Hypothetical and unknown |
| FTL_1223 | Unknown | Hypothetical and unknown |
| FTL_1224 | Thioredoxin | Energy metabolism |
| FTL_1251 | H+ dependent oligopeptide transport | Transport and binding proteins |
| FTL_1303 | *rpmE* | Protein synthesis |
| FTL_1306 | Unknown | Hypothetical and unknown |
| FTL_1402 | ISFtu1 transposase | Mobile and extrachromosomal element functions: |
| FTL_1503 | Deoxyguanosinetriphosphate triphosphohydrolase | Purines, pyrimidines, nucleosides, and nucleotides: |
| FTL_1511 | Glycerophosphoryl diester phosphodiesterase | Fatty acid and phospholipid metabolism |
| FTL_1790 | Major facilitator transporter | Transport and binding proteins |
| FTL_1812 | *hemE* | Biosynthesis of cofactors, prosthetic groups, and carriers |
| FTL_1832 | Siderophore biosynthesis | Transport and binding proteins |

**Table S4: Genes up-regulated at both the 4 and 8-hour time points**
