# Supplementary material for: Use of a Capture-Based Pathogen Transcript Enrichment Strategy for RNA-Seq Analysis of the Francisella Tularensis LVS Transcriptome during Infection of Murine Macrophages
Source: PLoS One. 2013 Oct 14;8(10):e77834. doi: 10.1371/journal.pone.0077834 (PMC3796476; doi:10.1371/journal.pone.0077834)
Supplement: Table S5 — Genes down-regulated at both the 4 and 8-hour time points. (DOC) [file pone.0077834.s005.doc]

| **Gene ID** | **Name/Function** | **Category** |
| --- | --- | --- |
| FTL_0004 | ISFtu1 transposase | Mobile and extrachromosomal element functions |
| FTL_0074 | Peptide deformylase | Protein Fate |
| FTL_0075 | Riboflavin synthase – beta subunit | Biosynthesis of cofactors, prosthetic groups, and carriers |
| FTL_0076 | Riboflavin synthase – alpha subunit | Biosynthesis of cofactors, prosthetic groups, and carriers |
| FTL_0227 | Ribozome recycling factor | Protein synthesis |
| FTL_0237 | *rplD* | Protein synthesis |
| FTL_0238 | *rplW* | Protein synthesis |
| FTL_0239 | *rplB* | Protein synthesis |
| FTL_0240 | *rpsS* | Protein synthesis |
| FTL_0241 | *rplV* | Protein synthesis |
| FTL_0243 | *rplP* | Protein synthesis |
| FTL_0244 | 50S ribosomal protein L29 | Protein synthesis |
| FTL_0249 | *rpsN* | Protein synthesis |
| FTL_0267 | Heat shock protein 90 | Protein Fate |
| FTL_0430 | Phospholipase | Fatty acid and phospholipid metabolism |
| FTL_0472 | DNA polymerase III – alpha subunit | DNA metabolism |
| FTL_0485 | *glgC* | Energy metabolism |
| FTL_0596 | UDP-glucose/GDP-mannose dehydrogenase | Energy metabolism |
| FTL_0599 | Glycosyl transferase | Protein Fate |
| FTL_0600 | Asparagine synthase | Amino acid biosynthesis |
| FTL_0601 | Sugar transamine/perosamine synthase | Cell envelope |
| FTL_0602 | Formyltransferase | Cell envelope |
| FTL_0799 | Type IV pili lipoprotein | Hypothetical and unknown |
| FTL_0828 | Type IV pili nucleotide binding protein | Protein Fate |
| FTL_0831 | Cyanophycin synthase | Biosynthesis of cofactors, prosthetic groups, and carriers |
| FTL_0891 | Peptidyl-prolyl cis/trans isomerase | Protein Fate |
| FTL_0892 | *clpP* | Protein Fate |
| FTL_0893 | *clpX* | Protein Fate |
| FTL_0894 | DNA-binding ATP-dependent protease | Protein Fate |
| FTL_0904 | SPFH-domain containing protein | Protein Fate |
| FTL_0905 | Phosphotyrosine protein phosphatase | Central intermediary metabolism |
| FTL_0906 | *engB* | Cellular processes |
| FTL_0929 | Unknown | Hypothetical and unknown |
| FTL_0964 | *hslU* | Protein Fate |
| FTL_0965 | ATP-dependent protease peptidase | Protein Fate |
| FTL_1050 | *rpoD* | Transcription |
| FTL_1128 | Unknown | Hypothetical and unknown |
| FTL_1190 | *grpE* | Protein Fate |
| FTL_1198 | *pheT* | Protein synthesis |
| FTL_1392 | Cold-shock DEAD-box protein A | Cellular processes |
| FTL_1542 | Unknown | Hypothetical and unknown |
| FTL_1610 | ISFtu1 transposase | Mobile and extrachromosomal element functions |
| FTL_1796 | ATP synthase – gamma subunit | Energy metabolism |
| FTL_1809 | i*nfB* | Protein synthesis |
| FTL_1810 | *nusA* | Transcription |
| FTL_1912 | *rpsA* | Hypothetical and unknown |
| FTL_1966 | Anthranilate synthase component I | Biosynthesis of cofactors, prosthetic groups, and carriers |

**Table S5: Genes down-regulated at both the 4 and 8-hour time points**
